# Supplementary material for: A high-resolution mRNA expression time course of embryonic development in zebrafish
Source: eLife. 2017 Nov 16;6:e30860. doi: 10.7554/eLife.30860 (PMC5690287; doi:10.7554/eLife.30860)
Supplement: Supplementary file 6. [file elife-30860-supp6.zip › biolayout-clusters-files/Cluster204-genes.html]

Cluster204


# Cluster204: Genes

| Ensembl ID | Gene Name | Chr | Start | End | Biotype |
| --- | --- | --- | --- | --- | --- |
| ENSDARG00000038834 | etfdh | 14 | 49083115 | 49115450 | protein\_coding |
| ENSDARG00000103740 | fundc2 | 21 | 44563677 | 44570464 | protein\_coding |
| ENSDARG00000054438 | fzd2 | 3 | 31490060 | 31492499 | protein\_coding |
| ENSDARG00000017803 | gsk3b | 9 | 9599706 | 9693207 | protein\_coding |
| ENSDARG00000053753 | mff | 15 | 35368704 | 35394579 | protein\_coding |
| ENSDARG00000018593 | parp16 | 25 | 31457307 | 31471657 | protein\_coding |
| ENSDARG00000009685 | ppa2 | 1 | 25695439 | 25704647 | protein\_coding |
